# Long-Term Response of Pembrolizumab in a Patient with Metastatic Squamous Non-Small Cell Lung Cancer on Hemodialysis: Case Report and Review of the Literature

**DOI:** 10.3390/medicina59020325

**Published:** 2023-02-09

**Authors:** Jae Won Yun, Jung Kwon, Taekyu Lim

**Affiliations:** 1 Veterans Medical Research Institute, Veterans Health Service Medical Center, Seoul 05368, Republic of Korea; 2 Division of Hematology-Oncology, Department of Internal Medicine, Veterans Health Service Medical Center, Seoul 05368, Republic of Korea

**Keywords:** pembrolizumab, hemodialysis, non-small cell lung cancer, end-stage renal disease, chronic kidney disease

## Abstract

In patients with renal failure and hemodialysis, there are difficulties in drug selection and dose adjustment for cancer treatment. The use of immune checkpoint inhibitors (ICIs), including pembrolizumab, approved by the U.S. Food and Drug Administration (FDA) for patients with metastatic non-small cell lung cancer (NSCLC) in 2015, has become an important option for the treatment of metastatic NSCLC. However, data regarding the dosage and schedule for long-term use of ICIs, especially pembrolizumab, in hemodialysis patients are limited. We present the case of a patient with metastatic squamous NSCLC who demonstrated a long-term partial response to pembrolizumab monotherapy for 45 months during hemodialysis and showed no immune-related adverse events (irAEs). To our knowledge, this is the longest remission to be reported without irAEs after discontinuation of pembrolizumab in a NSCLC patient undergoing HD. In addition, we reviewed previously reported lung cancer patients who used ICI during dialysis, comparing them with our case in clinical aspect. We believe that this report will provide clinical insights into the long-term efficacy and safety of pembrolizumab in lung cancer patients undergoing hemodialysis.

## 1. Introduction

Lung cancer (LCa) is one of the leading causes of cancer-related deaths worldwide [1]. The majority of patients are diagnosed with lung cancer at an older age, and it is often accompanied with other chronic diseases, including chronic kidney disease (CKD). The comorbidity prevalence of LCa patients was reported to be 43.3% in Sweden [2] and up to 87.3% in Scotland [3]. Comorbidities have been associated with delayed diagnosis and impaired performance status in patients with lung cancer. In particular, patients with end-stage renal disease (ESRD) and metastatic non-small cell lung cancer (NSCLC) undergoing hemodialysis have a poor prognosis and limited treatment options.

Pembrolizumab is a selectively humanized IgG4 kappa anti-PD1 monoclonal antibody that inhibits the PD-1 receptor on the surface of cytotoxic T lymphocytes. Previously, Kwok et al. [4] reported that pembrolizumab showed a dose-dependent increase in plasma concentration and that the clearance was positively correlated with body weight. Therefore, weight-based dosing compensates for different exposures owing to different weights. When positively correlated with body weight, clearance reaches steady-state pembrolizumab levels at 18 weeks in a 3-week dosing regimen. The clearance of pembrolizumab was reportedly not affected by either renal dysfunction or mild liver dysfunction [4].

The KEYNOTE-001 trial, conducted among 495 NSCLC patients treated with pembrolizumab, showed an overall response rate (ORR) of 19.4% (24.8% in the treatment-naïve group and 18% in the previously treated group. Notably, the ORR was not affected by histology, dose, or schedule of drug administration. The median duration of response, progression-free survival (PFS), and overall survival (OS) were 12.5 months, 3.7 months, and 12 months, respectively. In cases with PD-L1 expression in more than 50% of cancer cells, pembrolizumab demonstrated superior treatment efficacy [5]. In 2015, pembrolizumab was approved by the US Food and Drug Administration (FDA) for the treatment of metastatic NSCLC expressing PD-L1 that showed disease progression during or after platinum-based chemotherapy. Although pembrolizumab is not related to renal clearance, there are limited data and case reports describing the efficacy and safety of pembrolizumab in NSCLC patients who are undergoing hemodialysis due to CKD or ESRD.

Herein, we report a case of sustained partial response with no immune-related adverse events to pembrolizumab monotherapy in a patient with advanced squamous NSCLC and ESRD who was undergoing hemodialysis (HD).

## 2. Case Report

The patient was a 66-year-old man who was a former smoker (height, 167 cm; weight, 68 kg; and body surface area, 1.78 m^2^) with a 5-year history of CKD (serum creatinine, 2.3 mg/dL) due to IgA nephropathy. He had undergone a right middle and lower bilobectomy for moderately differentiated squamous cell lung cancer (cT1bN1M0, UICC/AJCC, 7th edition) in July 2012. The pathological stages were pT1bN1M0 and stage IIA according to the TNM classification of the UICC/AJCC, 7th edition. According to the UICC/AJCC 8th edition, it was classified as pT1cN1M0, stage IIB. PD-L1 expression was assessed using an immunohistochemistry assay (Dako, Carpinteria, CA, USA) with the murine 22C3 anti-human PD-L1 antibody, and the tumor proportion score (TPS) was 80%.

At the patient’s request, adjuvant chemotherapy was not administered. In March 2013, two small metastatic nodules (<1 cm) were found in the left upper and lower lobes. The patient refused further treatment and opted to be monitored closely until the symptoms appeared or changed. In March 2017, his renal function deteriorated, and since then he has been undergoing twice-weekly four-hour maintenance HD therapy.

In July 2017, a computed tomography (CT) scan of the chest revealed progression with left upper lobe atelectasis. The patient subsequently underwent four cycles of intravenous gemcitabine (D1, D8) every 3 weeks. According to the Response Evaluation Criteria in Solid Tumors (RECIST) version 1.1, stable disease was achieved, but he presented with progressive disease (PD) at 4 months.

**Figure 1:**
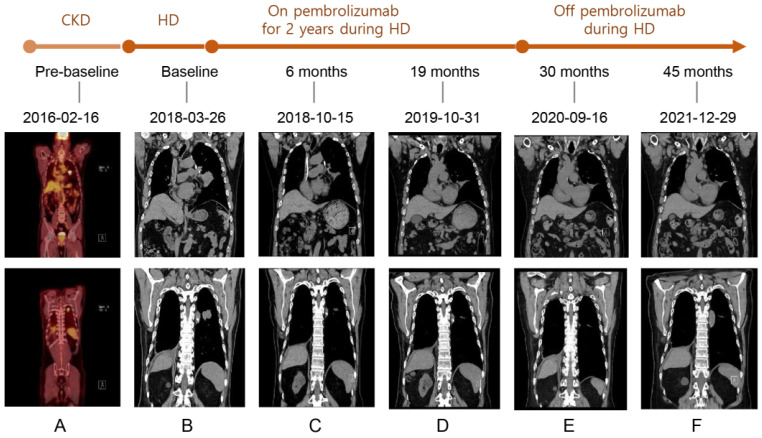
[F-18]FDG PET/CT scan and serial CT scans of the chest before and after pembrolizumab administration. (**A**) The [F-18]PET/CT scan on 16 February 2016 showed [F-18]FDG uptake in metastatic nodules in left upper lobe and left lower lobe. (**B**–**F**) The CT scans serially showed decreased extent of metastases in left lower lobe and left lower lobe from 26 March 2018 to 29 December 2021.

**Figure 2:**
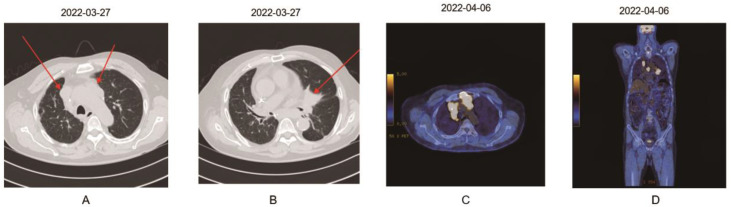
CT scan of the chest and [F-18]PET/CT scan on progression. (**A**,**B**) The CT scan of the chest on 22 March 2022 showed increased size of an increasing mass in central area of left upper lobe and enlarged LNs, as well as several new small nodules in both lungs. (**C**,**D**) The [F-18]PET/CT scan on 6 April 2022 showed [F-18]FDG uptake in metastatic nodules in the left and right upper lobes.

The patient subsequently opted for systemic therapy with pembrolizumab (200 mg/body) every three weeks from 26 March 2018. After six cycles of pembrolizumab administration, a CT scan demonstrated significant tumor shrinkage and confirmed a partial response (PR), according to RECIST. Pembrolizumab monotherapy was continued for 34 cycles through 11 March 2020, throughout HD, with no immune-related adverse events (irAEs). The antitumor effect was maintained with PR until 29 December 2021 (Figure 1). Three months later, he visited the emergency room for hemoptysis. A chest CT and a positron emission tomography-CT [PET-CT] scan showed progression, with a new lesion in the right upper lobe and enlargement of the mediastinal lymph nodes (Figure 2). On 8 April 2022, the patient, along with his family, decided he only wanted palliative care going forward. The patient died from respiratory arrest caused by hemoptysis.

## 3. Discussion

The high prevalence (12–53%) of CKD in cancer patients at diagnosis restricts their access to clinical trials and treatment options, potentially affecting their outcomes. Notably, in the study reported by Sprangers et al. (2020), serum creatinine thresholds were the exclusion criterion in 62% of the patients [6]. For this reason, there is a lack of the clinical data and experience regarding the long-term efficacy, safety, and adverse effects of ICI treatment in LCa patients undergoing dialysis. Recently, there were several reports of using ICI in LCa patients receiving dialysis [7,8,9,10], most of which were short-term follow-up cases of less than one year (Table 1). There was only one case in which a long-term clinical course was presented for more than 2 years after ICI treatment in LCa patients undergoing dialysis (Table 1), but the clinical details were provided in limited way [10]. Notably, all three cases using pembrolizumab apart from this case described adverse events or ICI discontinuation due to the adverse event (Table 1) [7,9,10]. To our knowledge, we report the first case of a pembrolizumab-treated LCa patient undergoing hemodialysis with a 45-month partial response and no adverse effects. Our study offers important clinical insights into the effective and safe long-term use of pembrolizumab in dialysis patients with LCa.

Several considerations need to be taken into account regarding cancer chemotherapy for this subset of patients. First, in ESRD patients, considering drug safety is crucial due to the increased risk of accumulation and toxicity. In the CANcer and DialYsis (CANDY) study, at least one drug needed dosage adjustment in 72% of dialysis patients on anticancer chemotherapy. In addition, a significant number of chemotherapeutic agents lack recommendations for dialysis patients [11]. The prescribing information on pembrolizumab states that renal function does not affect drug clearance, but no dose-timing guidance is provided for dialysis patients. No pharmacokinetic studies or experience from the manufacturer are available for dialysis patients [12]. However, case reports have shown that pembrolizumab can be safely administered to patients with ESRD [8].

Second, consideration of potential drug efficacy reduction due to ultrafiltration is necessary in dialysis patients. In the CANDY study, drug administration following dialysis was needed in 82% of dialysis patients receiving chemotherapy [11]. However, studies regarding other similar-sized antibodies have shown that they are not dialyzable because of their high molecular weight [8]. Therefore, it seems likely that pembrolizumab can be administered regardless of dialysis timing.

Third, patients with ESRD are considered immunocompromised [13], and renal failure has been reported to be associated with T-cell exhaustion and follicular helper T-cell imbalance [14]. T-cell inhibition and the immunosuppressive microenvironment of the tumor site result from the interaction between the PD-1 receptor and PD-L1. PD-1 antibodies disrupt this interaction, enabling the antitumor activity of T-cells [15]. Despite this immunological background of ESRD, T-cell exhaustion, and PD-1 roles, our case suggests that the PD-1 antibody, pembrolizumab, is still effective in dialysis patients. Further detailed investigations into the biological mechanisms involved in drug response in dialysis patients need to be performed.

In this report, pembrolizumab was administered without dose reduction (200 mg/body) based on the results that renal function did not affect pembrolizumab clearance in population pharmacokinetic studies and that the package insert did not recommend dose adjustments for CKD [4]. Due to its high molecular weight, pembrolizumab is unlikely to be cleared by dialysis. However, most patients with CKD are excluded from clinical trials, even if the drugs are not excreted by the kidneys [16]. Therefore, case reports and well-designed studies on the treatment of ICI in CKD should be performed in the future.

## 4. Conclusions

To our knowledge, this is a unique report of the longest remission without irAEs after discontinuation of pembrolizumab in a patient with metastatic NSCLC and ESRD who was undergoing HD. Further studies are needed to accurately determine the efficacy and safety of pembrolizumab in patients with NSCLC and ESRD undergoing HD. Clinicians should document and integrate evidence on the use of these agents in patients receiving HD.

## Figures and Tables

**Table 1:** Six lung cancer patients previously reported to have used ICI during dialysis.

No	Age (Year) /Sex	Type of Lung Cancer (Stage)	ICI (Dosage and Duration)	Underlying Kidney Disease	Dialysis Duration after Starting ICI (mo)	TR	PFS (mo)	OS (mo)	Adverse Events	Reference
1	66/male	Non-small cell lung cancer (IVB)	Pembrolizumab (~15 doses; 200 mg every 3 weeks)	ESRD due to diabetes mellitus	~11.3	PR	11.3	NA	Grade 1 rash	Ishizuka et al. [7]
2	72/male	Non-small cell lung cancer- adenocarcinoma (IIIB)	Pembrolizumab (discontinued after a single dose of 200 mg)	NA	12.5	CR	>12.5	>12.5	Mild ileus and aspiration pneumonia (suspected)	Osa et al. [9]
3	63/male	Squamous cell carcinoma of lung (IV)	Nivolumab (3 doses; 200 mg every 2 weeks)	Membrano-proliferative glomerulo-nephritis and monoclonal nephropathy	~2.4	SD	2.3	2.4	None	Jain et al. [8]
4	NA	NA	Atezolizumab (12 doses; 1200 mg every 3 weeks)	NA	12	NA *	<9	<12	None	Strohbehn et al. [10]
5	NA	NA	Pembrolizumab (34 doses; 200 mg every 3 weeks)	NA	~26	NA *	>26	>26	Hypothyroidism	Strohbehn et al. [10]
6	66/male	Squamous cell carcinoma of lung (initially IIB and progressed)	Pembrolizumab (34 doses; 200 mg every 3 weeks)	CKD due to IgA nephropathy	49	PR	45	49	None	This report

* Probably complete response, partial response, or stable disease (Data were not provided). Abbreviations: ESRD, end stage renal disease; CKD, chronic kidney disease; TR, tumor response; SD, stable disease; PR, partial response; CR, complete remission; PFS, progression free survival; OS, overall survival; ICI, immune checkpoint inhibitor.

## Data Availability

Not applicable.
